# An Experimental Comparison of Two Methods on Photosynthesis Driving Soil Respiration: Girdling and Defoliation

**DOI:** 10.1371/journal.pone.0132649

**Published:** 2015-07-15

**Authors:** Yanli Jing, Dexin Guan, Jiabing Wu, Anzhi Wang, Changjie Jin, Fenghui Yuan

**Affiliations:** 1 State Key Laboratory of Forest and Soil Ecology, Institute of Applied Ecology, Chinese Academy of Sciences, Shenyang, China; 2 Shenyang Agricultural University, Shenyang, China; Fudan University, CHINA

## Abstract

Previous studies with different experimental methods have demonstrated that photosynthesis significantly influences soil respiration (R_S_). To compare the experimental results of different methods, R_S_ after girdling and defoliation was measured in five-year-old seedlings of *Fraxinus mandshurica* from June to September. Girdling and defoliation significantly reduced R_S_ by 33% and 25% within 4 days, and 40% and 32% within the entire treatment period, respectively. The differential response of R_S_ to girdling and defoliation was a result of the over-compensation for R_S_ after girdling and redistribution of stored carbon after defoliation. No significant effect on RS was observed between girdling and defoliation treatment, while the soluble sugar content in fine roots was higher in defoliation than in girdling treatment, indicating that defoliation had less compensation effect for R_S_ after interrupting photosynthates supply. We confirm the close coupling of R_S_ with photosynthesis and recommend defoliation for further studies to estimate the effect of photosynthesis on R_S_.

## Introduction

Soil respiration (R_S_) returns 80.4 Pg carbon (C) back to the atmosphere annually [1], and represents the second largest carbon flux after photosynthesis in terrestrial ecosystem [2–3]. Therefore, even minor changes in R_S_ could have a large impact on atmospheric CO_2_ concentration. For this reason, studies on factors driving R_S_ have drawn much attention because of the need for accurately predicting terrestrial C budget and its possible feedback to climate change.

Soil temperature and soil moisture have been considered the main factors determining R_S_ and its underlying processes [4–5]. In recent years, however, a growing number of evidences have shown that photosynthesis supplying carbohydrates to roots and rhizosphere is a key driver of R_S_ [6–8]. Tight linkage between photosynthesis and soil respiration has been reported at diurnal, seasonal and annual time scales [9–15]. For example, R_S_ was consistent with diurnal pattern of the leaf photosynthetic substrate (soluble sugar and starch) content [16]. Similarly, annual R_S_ was significant correlated with gross primary production [9].

Many methods for evaluating photosynthesis effect on soil respiration have been employed, each having its own strengths and weaknesses [7]. For tree stands, girdling is a common interruption method which inhibits the flow of assimilates from leaves to roots, while enables water upward transport through the xylem. Results have indicated that girdling led to a significant decrease of 22% to 65% in R_S_ [3, 6, 17]. However, girdling is destructive and irreversible, and the increasing of root debris [18] and their symbionts after girdling may partly compensate or even over-compensate R_S_ because of microbial decomposition of dead roots [7, 12]. Nakane et al. [19] observed that dead root decomposition contributed amount to 20% of R_S_. For grassland or cropland ecosystems, defoliation is used to restrict the transportation of assimilates to belowground [20–25]. However, little information appears to be found on the response of R_S_ to defoliation in forest or seedlings.

As so many researches have been conducted in different ecosystems, it is practically important to compare the results from the two methods and causes of the discrepancies between them. Therefore, the aims of this study were to (1) compare the results of R_S_ in response to girdling and defoliation, and (2) put forward an appropriate method for future studies.

## Materials and Method

### Site description and experimental design

Measurements were carried out at the Research Station of Changbai Mountain Forest Ecosystem, Chinese Academy of Sciences (42°24′N, 128°05′E, and 738 m altitude) in northeastern China. The site has a temperate continental climate. The mean annual mean air temperature is 3.6°C, ranging from monthly temperature of -15.6°C to 19.7°C. The mean annual precipitation is 695 mm and about 80% precipitation occurs during the growing season [26–27].

Dark brown forest soil was collected from the top 20-cm in a near broadleaved korean pine mixed forest (described in detail in Table 1 [28]). *Fraxinus mandshurica* seedlings grown in a local nursery garden were transplanted in pots with dark brown forest soil inside in 2010. In early June 2013, twelve pots with seedlings (five-year-old, 1m height) were assigned randomly with a spacing of 1m×1m to avoid shading from each other. Control, girdling and defoliation treatments (each in four replicates) started on 22 June (leaf area index = 0.9 m^2^ m^-2^) and ended on 23 September (all leaves were fallen). The treatments were conducted as followed (1) girdling: completely removed over 5 cm wide sections of the trunk at 5 cm above ground, and (2) defoliation: removed all leaves of seedlings on 22 June, and defoliated other four times when leaf area exceeded 10 cm^2^. To better control soil moisture, a transparent shed was built 1m above the seedlings and all seedlings were irrigated at 18:00 h at about one-week interval.

**Table 1:** Physical-chemical properties on dark brown forest soil.

Total C (g kg^-1^)	Total N (g kg^-1^)	C:N ratio	Ca (μg g^-1^)	Mg (μg g^-1^)	Al (μg g^-1^)	PH
52.1	4.5	10.4	1841.4	267.9	15.1	5.5

### Soil respiration measurements

Soil respiration was measured by a soil CO_2_ efflux chamber connected to a LI-6400 portable photosynthesis system (LI-6400, LI-COR Inc., USA). Two PVC collars (11 cm in diameter and 5 cm in height) each pot were inserted to a depth of 2 cm into the soil one week before the first measurement and any living plants inside was removed. Measurements of soil respiration started on 2 June, 20 days before the treatments, to observe weather sample differences were existed. We then measured soil respiration of treatments from 22 June to 23 September at about 3-day interval. Measurements normally started at 8:30 a.m. and ended at 9:30 a.m. Continuous measurements were made on Jul. 6, Aug. 9 and Aug.31 at 2-h interval from 6:00 to 18:00 to monitor diurnal variation in soil respiration.

### Meteorological Measurements

Photosynthetic active radiation (LI-190SZ, LI-COR Inc., USA), air temperature and relative humidity (HMP45D, Vaisala, Finland) were all measured at 1.5 m height in a near meteorological station. Soil temperature at 10cm was measured with thermistor probe (109, Campbell Scientific inc., USA). Soil moisture at 10cm was measured by time domain reflectometry moisture meters (TDR200, Spectrum, USA). These data were logged every 10 min by a CR1000 (Campbell Scientific Inc., USA).

### Fine-root biomass and its non-structural carbohydrates

On September 23, all seedlings were harvested after soil respiration measurements and were partitioned into fine roots (≤2mm), coarse roots, stem and leaves. Fine roots were oven-dried at 80°C for about 48 h, and then weighed to determine their dry biomass and analyzed for sugar and starch using the anthrone method [29].

### Statistical analysis

One-way ANOVA was performed to compare effects of treatments on R_S_. Means were separated with Duncan’s test. The Q_10_ values were calculated according to *R*_*S*_ = ae^bt^ and Q_10_ = e^10b^ (where R_S_ is soil respiration (μmol m^-2^s^-1^), t is the soil temperature (°C), a is the basal respiration rate, and b is a constant). A significance level was set at P ≤ 0.05 and statistical analyses for all data were performed using SPSS 16.0 software package.

## Results

### Meteorological conditions

**Fig 1:**
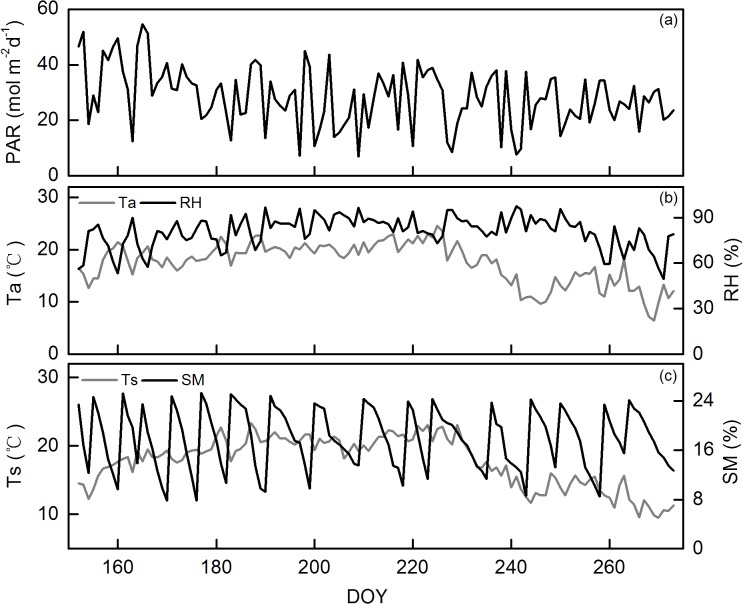
Seasonal variations of key meteorological variables in 2013, including daily cumulative photosynthetic active radiation (PAR) (a), daily average values of air temperature (Ta) and relative humidity (RH) (b), and daily average soil temperature (Ts) and volumetric soil moisture (SM) (c).

The seasonal variations of photosynthetic active radiation (PAR), air temperature and relative humidity, soil temperature and soil moisture are shown in Fig 1 (S1 File). Daily cumulative PAR peaked at a value of 54.6 mol m^-2^ d^-1^ in June, and then gradually decreased to 25.0 mol m^-2^ d^-1^ in September (Figure A in S1 File. The distribution of air temperature showed a large variation, ranging from 24.6°C on DOY 225 to 6.5°C on DOY 269 (Figure B in S1 File). Relative humidity was high during the whole study period, ranging from 49.4% to 97.5% (Figure B in S1 File). Soil temperature had similar pattern with air temperature, with the maximum of 23.4°C and the minimum of 9.5°C (Figure C in S1 File). The maximum soil moisture was 25.3% and the minimum was 7.8% (Figure C in S1 File).

### Seasonal variations in soil respiration

**Fig 2:**
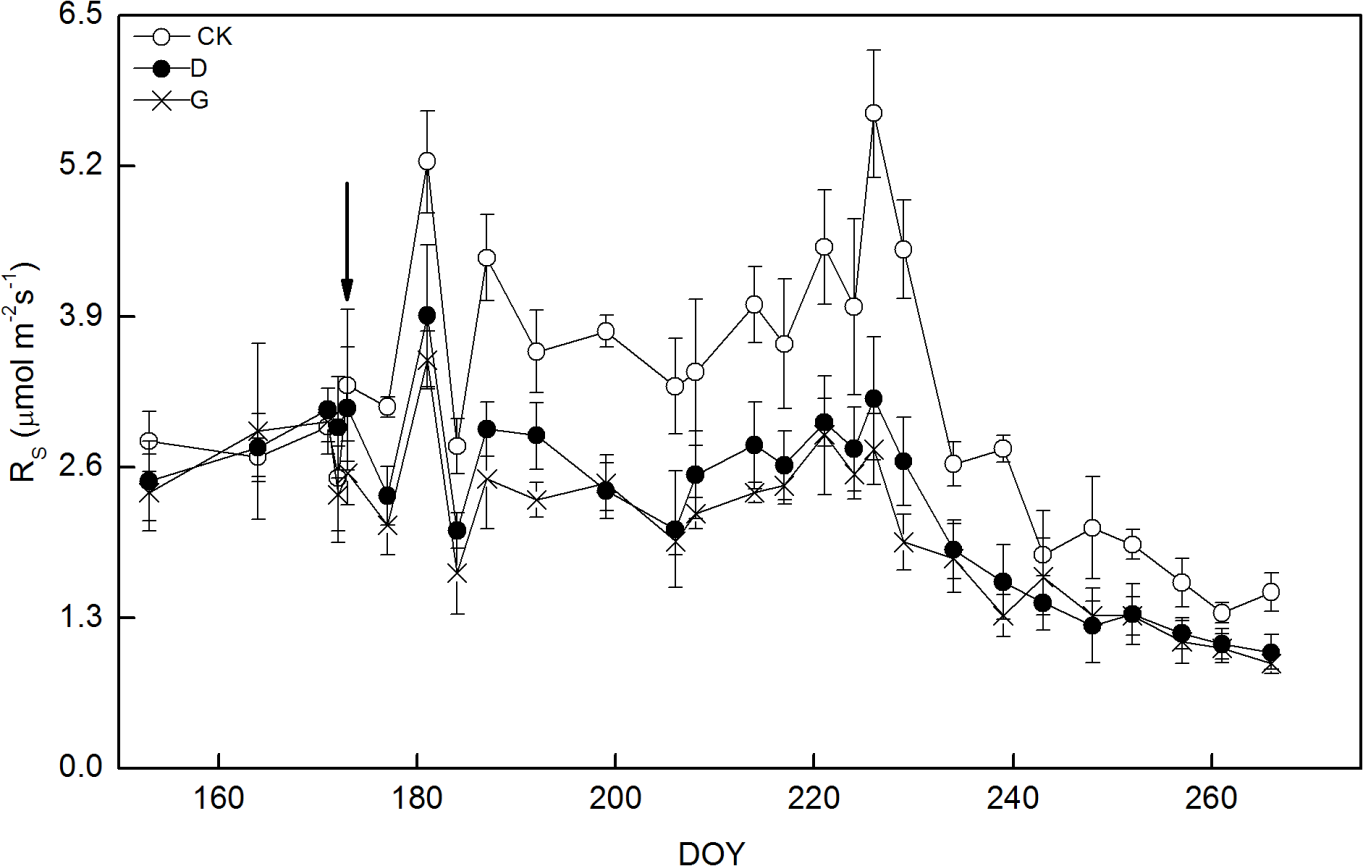
Seasonal variations of soil respiration in control (CK), girdling (G) and defoliation (D) treatments. Arrow shows the start of treatments and error bars represent standard deviation.

**Fig 3:**
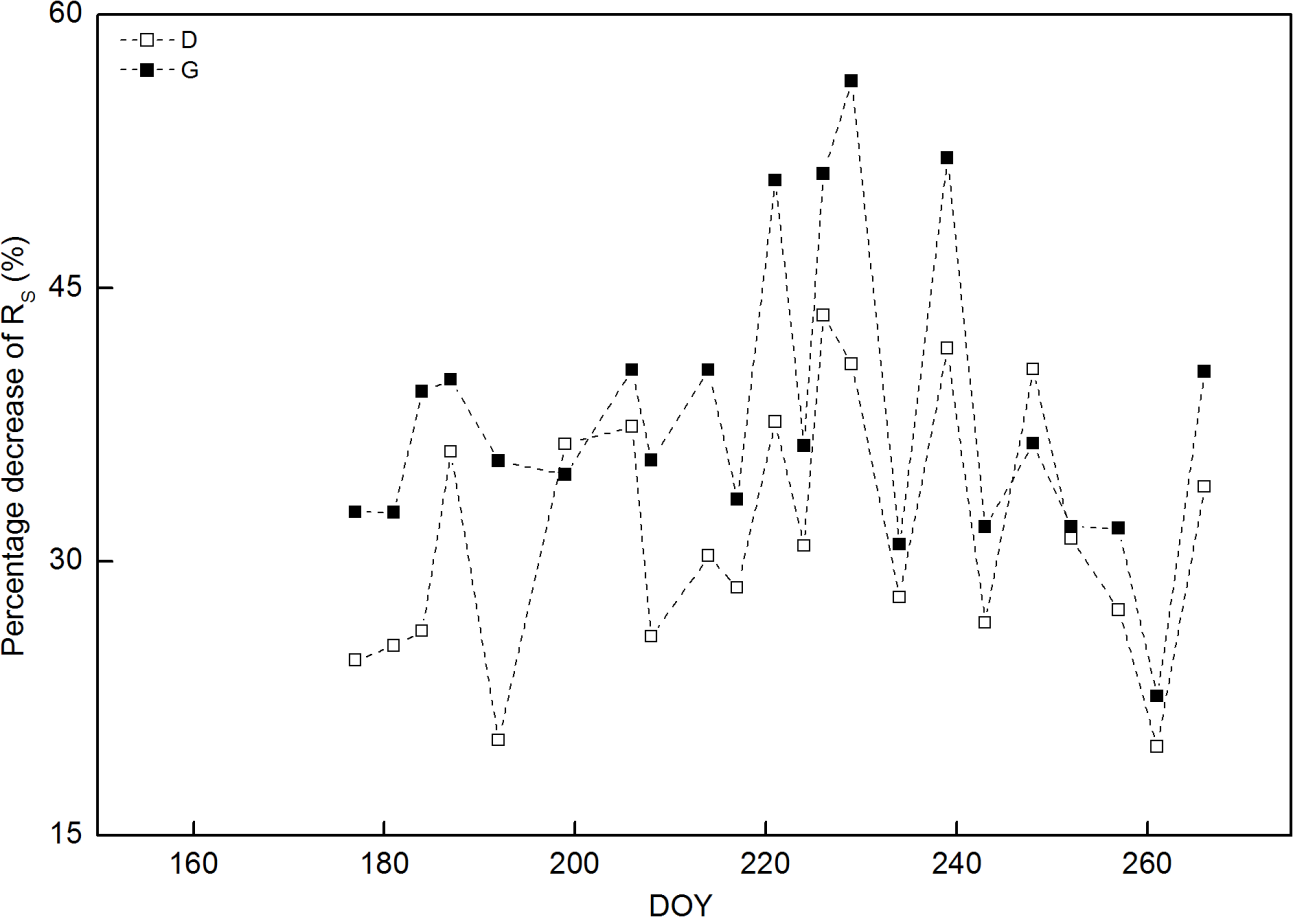
Seasonal variations of percentage decrease of soil respiration in girdling (G) and defoliation (D) treatments compared to the control.

R_S_ in controls followed a seasonal pattern, ranging from 1.3 μmol m^-2^ s^-1^ on DOY 261 to 5.7 μmol m^-2^ s^-1^ on DOY 226 (Fig 2) (S2 File). No significant difference in R_S_ was found among treatments during the pre-treatment period (DOY 153–173). However, R_S_ significantly decreased in girdling and defoliation treatments in comparison with the controls, while no significant difference in R_S_ was found between girdling and defoliation treatment during the treatment period (DOY 177–266). Within 4 days, R_S_ significantly decreased by 33% (P<0.01) and 25% (P<0.01) in girdling and defoliation treatments, respectively, relative to those measured in controls (Fig 3) (S3 File). These relative differences among treatments were fluctuant for the later 3 months, and decreased R_S_ reached its maximums of 56% (DOY 229, P<0.001) and 44% (DOY 226, P<0.01) in girdling and defoliation treatments, respectively. At the end of experiment, R_S_ was 40% (P<0.01) and 34% (P<0.01) lower in girdling and defoliation treatments than in controls, respectively. Overall, the mean R_S_ declined by 40% (1.94 vs. 3.22 μmol m^-2^ s^-1^) and 32% (2.18 vs 3.22 μmol m^-2^ s^-1^) in girdling and defoliation treatments compared to the controls during the treatment period, respectively.

### Diurnal variations in soil respiration

**Fig 4:**
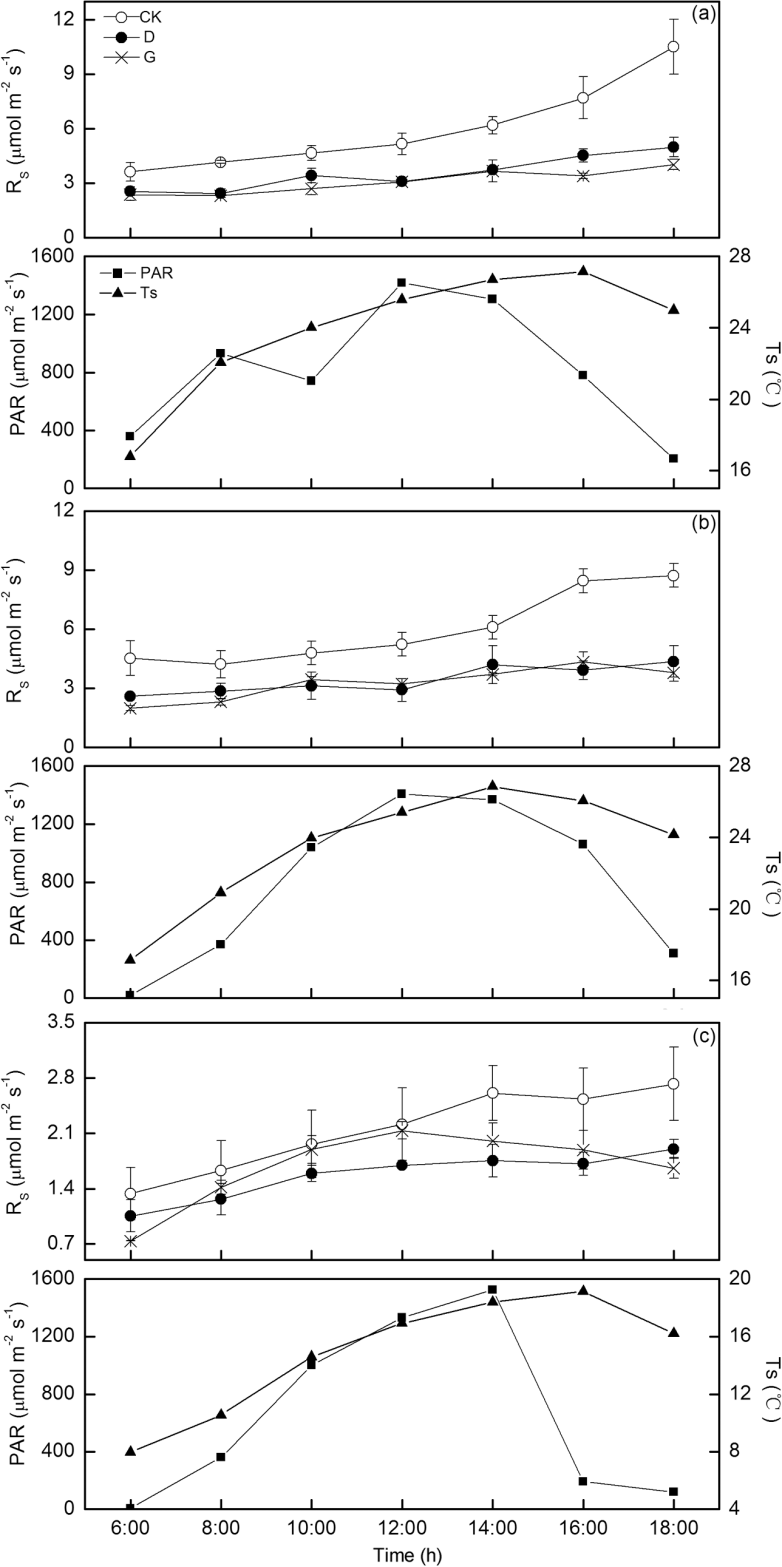
The diurnal patterns of soil respiration (R_S_) in the control (CK), girdling (G) and defoliation (D) treatments on Jul. 6 (a), Aug. 9 (b) and Aug. 31 (c) along with PAR and soil temperature. Error bars represent standard deviation.

The diurnal patterns of R_S_, soil temperature and PAR on Jul. 6, Aug. 9 and Aug.31 were shown in Fig 4 (S4 File). Generally, PAR peaked at 12:00–14:00 h, and peak values of soil temperature lagged 2 hours. In control, R_S_ dramatically increased since 14:00 h and reached the peaks at 18:00 h, with the values of 10.5, 8.7 and 2.7 μmol m^-2^ s^-1^ on Jul. 6 (Figure A in S4 File), Aug. 9 (Figure B in S4 File) and Aug.31(Figure C in S4 File), respectively. In girdling and defoliation treatments, however, R_S_ exhibited low and similar diurnal variations on Jul. 6 and Aug. 9. The maximum of R_S_ was 62% and 53% lower in girdling and defoliation treatment relative to the control on Jul. 6, which was 57% and 54% lower on Aug. 9, respectively. However, R_S_ showed higher diurnal variations and mean in girdling treatment than in defoliation treatment on Aug.31.

### Temperature response of soil respiration

There were significant exponential relationships between R_S_ and soil temperature in the control (R^2^ = 0.86, P<0.001), girdling (R^2^ = 0.75, P<0.001) and defoliation (R^2^ = 0.80, P<0.001) treatments, respectively (Table 2) (S5 File). The coefficient a (basal respiration) was 0.6115, 0.4642 and 0.419 in control, girdling and defoliation treatments, respectively. Girdling and defoliation declined basal respiration by 24% (P<0.01) and 31% (P<0.01), respectively. The coefficient b was lower in girdling (0.073±0.008) and defoliation (0.083±0.006) treatments than in controls (0.085±0.002). Consequently, Q_10_ was suppressed by 11% (P<0.05) and 2% (P>0.05) due to girdling and defoliation, respectively.

**Table 2:** Values of coefficients a and b of the Eq. (*R*_*S*_ = *ae* ^*bT*^), the temperature sensitivity of soil respiration (Q_10_) and their one-way ANOVA test among different treatments.

Treatment	a	b	Q_10_	R^2^	P
Control	0.6115±0.05a	0.085±0.002a	2.35±0.05a	0.86	<0.001
Girdling	0.4642±0.08b	0.073±0.008b	2.08±0.17b	0.75	<0.001
Defoliation	0.4190±0.04b	0.083±0.006a	2.30±0.13a	0.80	<0.001

A one-way ANOVA was used to compare a, b and Q_**10**_ values among different treatments. Different letters mean significant difference among treatments at P< 0.05 (Mean±SD, n = 4).

### Fine-root biomass and its non-structural carbohydrates

The fine-root biomass was significantly lower (P<0.01) in girdling (8.3±0.8) and defoliation (8.6±0.9) treatments than in the control (46.2±2.1) (Table 3) (S6 File). The soluble sugar content in fine roots was 83% (P<0.001) and 48% (P<0.001) lower in girding and defoliation treatments than in controls, respectively. The starch content in fine roots significantly declined by 74% (P<0.001) and 73% (P<0.001) in the girdling and defoliation treatments, respectively. Significant difference in soluble sugar content was found between defoliation and girdling treatment, but not in starch content or in fine- root biomass.

**Table 3:** Fine-root biomass and its soluble sugar and starch contents among different treatments.

Treatment	biomass (g)	soluble sugar content (mg/g)	starch content (mg/g)
Control	46.2±2.1a	32.5±1.6a	46.6±5.6a
Girdling	8.3±0.8b	5.7±0.1c	11.7±2.2b
Defoliation	8.6±0.9b	17.1±2.6b	11.8±2.0b

A one-way ANOVA was used to compare biomass, soluble sugar content, and starch content in fine-root among different treatments. Different letters mean significant difference among treatments at P< 0.05 (Mean±SD, n = 4).

## Discussion

### Effect of girdling and defoliation on soil respiration

It was reported that soil respiration in girdling plots declined by 37% within 5 days [6] and 53% within two months [12]. This is similar with our results that girdling resulted in a significant decrease of soil respiration by 33% within 4 days and 56% on DOY229, respectively (Figs 2 and 3). The rapid decline in soil respiration following girdling supports previous findings [30–31] that C recently assimilated by plant plays an important role in driving soil respiration. In the current study, soil respiration decreased by 40% (on average) due to girdling, which is similar with 50% reduction of soil respiration reported in previous girdling studies [3, 32–33]. Little reduction of total soil respiration (14 and 27%) after 8-month girdling was reported in *A*.*crassicarpa* and *E*.*urophylla* plantations and no change in soil respiration in response to girdling for the first 6 weeks after girdling was found in spruce; these results were attributed to the large carbohydrate reserves in the roots [34–35]. However, the reduction of soil respiration was underestimated after girdling, because: (1) the microbial decomposition of dead roots caused by girdling may enhance heterotrophic respiration [7, 31], and (2) the stored carbohydrates in roots may be consumed in the girdling treatment [12, 18]. In this study, fine-root biomass and non-structural carbohydrates in fine roots were significantly suppressed after 3-month girdling (Table 3). This confirms the underestimation of photosynthesis influencing soil respiration after girdling and indicates that stored carbon in belowground plays a significant role in compensation for the carbon loss of soil. The underestimation has been found in previous studies [6, 12, 17–18, 30–31], however, to our knowledge, no study has eliminated the underestimation using interrupting photosynthetic methods.

Previous studies have proven that defoliation reduced the allocation of the net assimilated carbon to below ground [22, 36], resulting in a decrease in root biomass [20–21, 37] as well as non-structural carbohydrates [21] and an increase in soil microbial biomass [25, 38]. In the current study, defoliation significantly decreased soil respiration by 32%, including the compensation from stored carbon in roots and microbial decomposition of dead roots. This contrasts with the findings of Snyder and Williams, who reported no defoliation effect on root respiration in *Populus fremontii* saplings [39]. The differences between these two studies were possibly because (1) continuous defoliation in this study inhibited photosynthates supplying to roots, while half of the leaves left in *Populus fremontii* saplings did not, and (2) 3.5 times larger in root biomass of *Populus fremontii* saplings than this study maintained its respiration after defoliation.

### Comparison of the effect of girding on soil respiration with defoliation

Similar diurnal patterns (on Jul. 6 and Aug. 9) and seasonal patterns in soil respiration were observed in girdling and defoliation treatments in the current study. This indicated that girdling and defoliation are both effective methods for interrupting the flow of recent photosynthates to the roots. However, higher diurnal variations and mean in soil respiration were found on Aug. 31 in girdling treatment than in defoliation treatment. According to Zeller et al. [40], microbial populations were larger in the girdled plots than the control ones after 2-month girdling. Therefore, this is likely the time at which new source of carbon through the decomposition of decaying root material peaked in girdling treatment and over compensated for soil respiration. However, soil respiration was not continuously measured and the seasonal variations of compensation effect were failed to estimate in this study. Thus, more clearly demonstration of carbon dynamics is needed to explain seasonal variation of soil respiration after girdling. In addition, soil respiration decreased more in girdling treatment (by 40%) compared to defoliation treatment (by 32%) in this study. Piper and Fajardo suggested that regrowth ranged from 19% of initial leaf biomass to 42% after the second complete defoliation [41]. In this study, other 4 times defoliations were conducted after the first defoliation. Thus, the regrowth of leaves led to a decrease of stored carbon and an increase of photosynthates supply for roots, which partly weakened the reduction of soil respiration in defoliation treatment.

### Advantages and disadvantages of the two methods

Girdling is a widely used method for forest, which is simple, cheap, requires no expensive analyses and causes little disturbance in soil moisture and temperature [3, 12, 31]. However, decomposition of decaying root material after girdling compensated or over- compensated for soil respiration. Earlier studies have documented that evergreen and deciduous species have different storage strategies of carbon [41–42]. For example, *Fraxinus mandshurica* stored more non-structural carbohydrate in belowground than *Larix gmelinii* [43]. Thus, the underestimation of photosynthesis influencing soil respiration following girdling may be magnified for evergreen species. Krause et al. [44] indicated that starch content of coarse roots in girdled tree began to decrease after 4-week later and root starch was almost depleted after 10-month later in a mature Norway spruce stand. In the current study, 3 months later, root sugar and starch significantly declined in girdled seedlings (Table 3). This suggests that short-term (days) decrease of soil respiration caused by girdling derives from interrupting recent photosynthates, while long-term (months) response of soil respiration to girdling is combined a negative effect of recent photosynthates with a positive effect of stored carbon used by microbes.

Defoliation has similar advantages to girdling, while it has less compensation effect than girdling. This is supported by higher soluble sugar content in fine roots in defoliation than in girdling treatment (Table 3). Therefore, defoliation method should been paid more attention to the effect of recent photosynthates and stored carbon in plants on soil respiration.

## Conclusion

Soil respiration has similarly responses to girdling and defoliation at daily and seasonal time scales. The rapid declines in soil respiration following girdling and defoliation within 4 days and 3 months suggest that the decreases in soil respiration are not simply a consequence of a reduced current photosynthates supply to roots; stored carbon also plays an important role. This conclusion is derived from the findings that fine-root biomass and non-structural carbohydrates in fine roots were significantly reduced after girdling and defoliation. Defoliation had less compensation effect than girdling. Therefore, defoliation method should been paid more attention in the further studies of photosynthesis driving soil respiration.

## Supporting Information

S1 File Seasonal variations of key meteorological variables in 2013, including daily cumulative photosynthetic active radiation (PAR) (Figure A in S1 File), daily average values of air temperature (Ta) and relative humidity (RH) (Figure B in S1 File), and daily average soil temperature (Ts) and volumetric soil moisture (SM) (Figure C in S1 File). (XLS)

S2 File Seasonal variations of soil respiration in control (CK), girdling (G) and defoliation (D) treatments. Arrow shows the start of treatments and error bars represent standard deviation.
(XLS)

S3 File Seasonal variations of percentage decrease of soil respiration in girdling (G) and defoliation (D) treatments compared to the control. (XLS)

S4 File The diurnal patterns of soil respiration (R_S_) in the control (CK), girdling (G) and defoliation (D) treatments on Jul. 6 (Figure A in S4 File), Aug. 9 (Figure B in S4 File) and Aug. 31 (Figure C in S4 File) along with PAR and soil temperature. Error bars represent standard deviation.
(XLS)

S5 File Data for Table 2. Values of coefficients a and b of the Eq. (*R*_*S*_ = *ae* ^*bT*^), the temperature sensitivity of soil respiration (Q_10_) and their one-way ANOVA test among different treatments.
(XLS)

S6 File Data for Table 3. Fine-root biomass and its soluble sugar and starch contents among different treatments.
(XLS)
